# Physical activity and sitting time prior to and during COVID-19 lockdown in Austrian high-school students

**DOI:** 10.3934/publichealth.2021043

**Published:** 2021-07-26

**Authors:** Klaus Greier, Clemens Drenowatz, Theresa Bischofer, Gloria Petrasch, Carla Greier, Armando Cocca, Gerhard Ruedl

**Affiliations:** 1 Division of Physical Education, Private Educational College (KPH-ES), Stams, Austria; 2 Department of Sports Science, University of Innsbruck, Innsbruck, Austria; 3 Division of Sport, Physical Activity and Health, University of Education Upper Austria, Linz, Austria

**Keywords:** moderate physical activity, vigorous physical activity, walking, sedentary behavior, movement restriction, adolescents

## Abstract

The COVID-19 pandemic has led in many countries to the implementation of policies that mandate social distancing and movement restrictions. While these measures are warranted in order to minimize the spread of the virus they may have detrimental effects on various behaviors, including physical activity (PA). The present study examined PA and sitting time in 14 to18-year-old Austrian high school students prior to and during the second COVID-19 lockdown in Austria. Data was collected via an online questionnaire during fall/winter 2020/21. Questions were based on the International Physical Activity Questionnaire, which examined frequency and duration of PA and sitting time. A total of 221 high school students provided valid data. Participants reported significantly lower moderate and vigorous PA during the lockdown while sitting time increased (p < 0.01). The frequency of walking (days/week) also decreased during COVID-19 lockdown, which also contributed to a significant decline in total walking time (p < 0.01). Further, the decline in PA was more pronounced in boys, while girls reported a greater decline in walking. These differences were due to higher PA and walking in boys and girls, respectively, prior to the lockdown. During the lockdown sex differences in PA and sitting time were limited. Taken together, these results highlight the impact of COVID-19 policies on PA in adolescents and emphasize the importance to promote an active lifestyle even in times of home confinement.

## 1. Introduction

The benefits of physical activity (PA) for the development of children and adolescents along with its impact on physical, mental and social health have been well documented [1],[2]. Nevertheless, PA levels and motor competence have declined in children and adolescents during the last several decades [3]–[5]. In Germany for example, only 27.5% of children and adolescents between 3 and 17 years of age have been shown to meet current PA recommendations of 60 minutes of moderate-to-vigorous PA [6].

A global pandemic caused by the new coronavirus (COVID-19) may further affect PA in children and adolescents due to the implementations of various restrictions on daily life that included social distancing and the closure of public spaces along with stay at home orders, which should mitigate the spread of the virus [7]–[9]. Among other aspects, these polices included closures of universities, schools, sports clubs and fitness centers. Even though these measures can help in controlling the spread of the virus, confinement at home and social distancing can have a significant impact on other correlates of public health. Various international studies showed a reduction in PA while sedentary behaviors and screen time increased during COVID-19 lockdowns [10]–[15]. Lockdown policies, however, differed in severity and duration between countries, which limits the comparability of studies. The majority of research also focused either on the adult population or pre-pubertal adolescents while research on the effects of COVID-19 policies in older adolescents has been limited. Various lifestyle habits, however, are established during late adolescence [16],[17] and even though lockdown policies are only temporary they may have a significant impact on behavioral choices in the future. Accordingly, limiting or preventing school and club sports as well as closures of indoor and outdoor sport facilities can have a significant impact on future PA and general health [18],[19].

After lifting movement restrictions in May 2020, Austria implemented a second lockdown from November 17, 2020 until January 18, 2021. Among other policies that emphasized social distancing, schools and club sports facilities were closed during these times. Even though movement restrictions were not as severe as in other countries [20], students were forced into online teaching, which also resulted in the cancellation of in-person physical education classes. Given the lack of research on the impact of Austrian COVID-19 policies on PA in high school students the present study examined differences in PA behaviors between the second COVID-19 lockdown and the period prior to the lockdown.

## 2. Materials and methods

A total of 5 high-schools in the city of Innsbruck, Austria were randomly selected for participation. One school declined to participate for administrative reasons, which resulted in 4 schools with roughly 900 eligible participants between 14 and 18 years of age. The study protocol was approved by the School Board of the Federal State of Tyrol, Austria and participants provided consent at the time of data collection.

Data was collected via an online questionnaire using SoSci Survey (SoSci Survey GmbH, Munich, Germany) during the second lockdown, between the end of November and mid-December 2020. In addition to information on age and sex, participants were asked about their PA and sedentary behavior during and prior to the lockdown. The period prior to the lockdown referred to the time between the beginning of the school year in mid-September until the start of the second lockdown on November 17. During that time, schools were open and students engaged in regular physical education (3 classes of 50 minutes per week). Also clubs sports and other sports facilities (e.g. swimming pools) were available to the public during this period.

The PA questions were based on the International Physical Activity Questionnaire—short form (IPAQ-SF), which provides information on time spent in moderate PA (MPA) and vigorous PA (VPA) as well as time spent walking and sitting [21]. Specifically, participants were asked to report the number of days they engaged in MPA, VPA and walking within one week and how much time they spent doing these activities on those days. Additionally, participants reported the average daily sitting time on a weekday during the pre-lockdown and lockdown period. The responses for MPA and VPA were subsequently categorized into 4 groups for the number of days per week (0–1 days/week, 2 days/week, 3 days/week, >3 days/week) and 3 groups for time spent (<1 hour/day, 1 hour/day, >1 hour/day), respectively. Walking time was categorized into 3 groups for the number of days (0–2 days/week, 3–4 days/week, >4 days/week) and duration (<1 hour/day, 1 hour/day, >1 hour/day). Sitting time was also stratified into 3 groups (<9 hours/day, 9 hours/day, >9 hours/day). Additionally, total time spent (hours/week) was calculated for MPA, VPA and walking. Subsequently change in these behaviors along with change in sitting time was calculated as time spent in the respective behavior during the lockdown minus the time spent in this behavior prior to movement restrictions.

*Statistical Analysis*. Differences between pre-lockdown and COVID-19 lockdown in the prevalences of time spent in VPA, MPA, walking and sitting time were analyzed via McNemar tests. In addition, one sample t-tests were used to examine behavioral changes in total time spent in various behaviors. Further, mixed between-within 2 (sex) x 2 (Pre vs. lockdown) ANOVAs were used to examine sex differences in behavioral changes. All statistical analyses were conducted with SPSS 26.0 with a significance level of p < 0.05.

**Table 1.** Physical activity and sitting time prior to and during COVID-19 confinement. Values are Prevalence (%).

	*Days/week*	Pre-Lockdown	COVID-19 Lockdown	*Hours/day*	Pre-Lockdown	COVID-19 Lockdown
Moderate PA	0–1 days/week	15.8	28.1	<1 hr/day	10.4	22.2
	2 days/week	32.1	35.7	1 hour/day	70.1	57.9
	3 days/week	32.6	27.6	>1 hour/day	19.5	19.9
	>3 days/week	19.5	8.6			
Vigorous PA	0–1 days/week	24.9	60.6	<1 hr/day	16.3	57.1
	2 days/week	26.2	23.1	1 hour/day	41.2	37.1
	3 days/week	33.0	13.6	>1 hour/day	42.5	5.9
	>3 days/week	15.8	2.7			
Walking	0–2 days/week	32.1	78.3	<1 hr/day	31.2	38.0
	3–4 days/week	33.9	19.5	1 hr/day	32.6	37.6
	>4 days/week	33.9	2.3	>1 hr/day	36.2	24.4
Sitting time				<9 hrs/day	24.4	0.0
				9 hrs/day	49.3	7.2
				>9 hrs/day	26.2	92.8

## 3. Results

**Figure 1.**
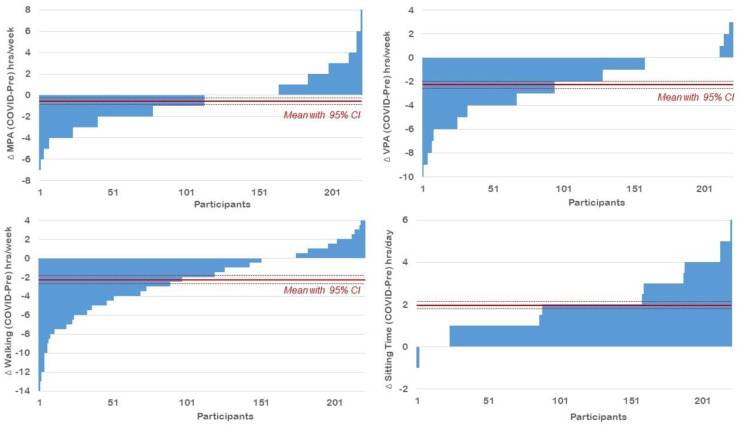
Individual change in physical activity, walking and sitting time from pre-COVID-19 to lockdown.

A total of 221 (51.1% male) high-school students with an average age of 15.7 ± 1.3 years provided valid data. Prior to the lockdown more than half of the participants (52.0%) engaged in MPA for at least 3 days/week and almost half of the participants (48.9%) engaged in VPA for at least 3 days/week. On these days a majority or participants reported more than 1 hour/day of PA (89.6% for MPA and 83.7% for VPA, respectively). More than 2/3 of the participants (67.9%) reported walking at least 3 days/week and almost ¼ (24.4%) had less than 9 hrs/days of sitting time (Table 1). With the COVID-19 lockdown there was a significant decline in the number of days participants engaged in MPA and VPA as well as walking (p < 0.01). Similarly, daily time spent in MPA and VPA was lower during COVID-19 confinement (p < 0.01), while no difference was observed for walking time. Nevertheless, total walking time decreased on average by 2.3 hours/week with COVID-19 restrictions (p < 0.01) as did total weekly time spent in MPA (−0.6 hours/week) and VPA (−2.3 hours/week) (p < 0.01). Sitting time, on the other hand, significantly increased by 2.0 hours/day during lockdown as compared to prior to the implementation of movement restrictions (p < 0.01) (Figure 1).

**Figure 2.**
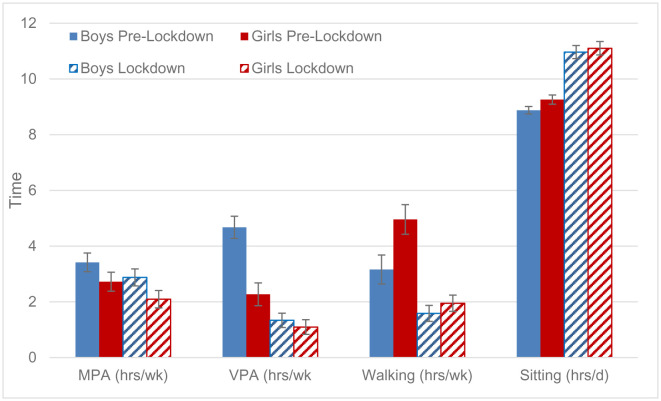
Physical activity and sitting time pre-lockdown and during COVID-19 lockdown, separately for boys and girls. Values are mean with 95% CI.

Behavioral changes, however, differed by sex as indicated by significant time by sex interaction effects for VPA and walking (p < 0.01). Prior to the lockdown, VPA was significantly higher in boys, while girls displayed higher walking time (p < 0.01). These differences were no longer significant during lockdown. Accordingly, the reduction in VPA was more pronounced in boys, while girls displayed a greater decline in walking. No significant interaction effect was observed for MPA. Even though boys reported higher MPA compared to girls prior to the lockdown, both, boys and girls, displayed a similar decline during lockdown. There was also no significant interaction effect for sitting time despite the fact that sitting time was higher in girls compared to boys prior to the lockdown (p < 0.01) and these differences were no longer significant during lockdown (Figure 2).

## 4. Discussion

The present study showed a decline in the amount and intensity of physical activity during the second COVID-19 lockdown in Austrian secondary school students, which was implemented during fall/winter 2020. Sitting time, on the other hand, was significantly higher during the lockdown as compared to prior to the implementation of movement restrictions. These results are consistent with previous studies that examined the effect of movement restrictions during the COVID-19 pandemic [10]–[12],[20],[22]–[24]. Interestingly, there was a consistent decline in the number of days across all forms of PA, while the decline in time spent per day was only significant for MPA and VPA but not for walking. The decline in PA may be attributed to the closure of sports clubs, fitness centers and public sports facilities during lockdown. Given the lack of available facilities, these adolescents were no longer able to continue their regular training schedule in various sports, which most likely affected total PA. The decline in walking frequency may be attributed to school closures and the transition to distance learning as well as home confinement in general, as these measures limited opporutnities for active transportation. During school closures, teaching also focused on the subjects of maths and languages as well as natural and social sciences. Physical education classes consisted of recommendations and suggestions for physical activities at home but the control of the engagement in these activities was limited.

Taken together, these results highlight the impact of the implemented policies on social distancing and movement restrictions on adolescents' PA, which can impact their health and well-being. The detrimental effects of insufficient PA on various health conditions in children and adolescents have been well documented [25]–[28]. Even though a lockdown may have been necessary to minimize the spread of a viral disease, such policies, may have some unintended consequences as chronic diseases remain a major threat to future public health [29],[30]. School closures and the loss of a daily structure along with social distancing can further affect psychological well-being as available data indicates an increase in depressive symptoms and anxiety in children and adolescents during the lockdown [31]–[33].

In addition to the decline in PA, participants also reported an increase in sitting time by 2 hours/day during lockdown. Besides the lack of opportunities for PA, this behavioral change can also be attributed to the engagement in distance learning as a result of school closures. Children were required to spend a lot of time in front of a computer in order to complete their school work. With limited opportunities for social interactions in a natural setting, social media presence, most likely, increased, which further facilitated high screen time and sedentary behavior. As has been shown for PA, these results are consistent with studies from other countries [13],[24],[34],[35]. Given the independent association of high sitting time with various health outcomes this may further increase the risk for future health problems [36],[37]. High sedentary time has detrimental effects on the development of cardio-vascular disease with an increased morbidity and mortality risk of daily sitting times exceeding 6 to 8 hours [38]; in the present study, adolescents reported at least 9 hours/day of sitting. The detrimental effects of high sitting time, however, can be mitigated by regular interruptions of sedentary behaviors [39],[40] and, therefore, should be emphasized in times of movement restrictions.

The significant impact of COVID-19 policies on behavioral choices is further indicated by the fact that sex differences in PA and sitting time declined during the lockdown. Given the higher activity levels in boys prior to the lockdown, they displayed a greater decline, particularly in VPA. Girls, on the other hand, displayed a more pronounced decline in walking as they spent more time walking compared to boys prior to the lockdown. The lack of sex differences in various behaviors may also be attributed to the lack of behavioral choices during home confinement as similar effects have been shown in adults, with a more pronounced decline in PA during COVID-19 in men compared to women [23].

Some limitations of the present study, however, need to be considered when interpreting the results. Physical activity and sitting time were assessed via questionnaire at a single time point. Even though this method has been commonly used, particularly in studies examining the effects of COVID-19 on behavioral choices, there is a risk of over-reporting and recall bias [41],[42]. Further, no information on additional behavioral choices (e.g. diet, sleep) as well as anxiety and stress were collected. The study population also consisted of a convenience sample that was limited to the city of Innsbruck and there was no information on socio-economic background and living situation. In a rural area, where people may have more opportunities for outdoor activities due to private yards or nearby walking trails results may differ. In conjunction with other studies, there is, however, strong evidence of detrimental effects of COVID-19 policies on PA [10]–[12],[22]–[24].

## 5. Conclusions

While the global spreading of COVID-19 warranted strong measures in order to limit the spread of the virus, the negative effects on other health-related aspects should not be overlooked. The detrimental effects of low PA and high sitting time on physical and cognitive development as well as general health and well-being have been well documented [43]–[45] and sufficient PA has also been associated with better protection against viral infections [46]. As various lifestyle habits are established during adolescence [16],[47], it is particularly important to provide opportunities for and emphasize the importance of PA at this age even in times of movement restrictions. Accordingly, guidelines for physical activities at home, such as those provided by the WHO [48], need to be promoted. It should also be ensured that adolescents return to a more active lifestyle once COVID-19 restrictions have been lifted in order to promote their future health and wellbeing.
